# The linker histone H1.4 condenses chromatin in maturing postmitotic neurons

**DOI:** 10.64898/2026.07.28.741367

**Published:** 2026-07-29

**Authors:** Andrew I. Aldridge, Martine W. Tremblay, Vijyendra Ramesh, Xiaolin Wei, Yong-Hui Jiang, Anne E. West

## Abstract

H1 histones are abundant nuclear proteins that bind to linker DNA at the entry and exit points of nucleosomes. Although individual H1 family members play partially redundant roles in chromatin organization, the discovery of disease-associated mutations in H1 genes has raised interest in their cell-type specific functions. Heterozygous, de novo frameshift mutations in *H1-4* cause the neurodevelopmental disorder Rahman Syndrome, which is characterized by mild to severe intellectual disability along with other neurological and morphological features. H1.4 has mostly been studied in dividing cells, and its expression in the brain was poorly understood. Here we characterized the expression of *H1f4* mRNA and H1.4 protein in the brains of male and female mice across postnatal development. Using an epitope-tagged *H1f4* knockin mouse, we show that this linker histone is robustly expressed throughout the brain including in mature, post-mitotic neurons of adult mice. By chromatin immunoprecipitation, we observe that H1.4 binds broadly across the genome in neural progenitors and accumulates in heterochromatin over the course of neuronal maturation. Finally we show that developmental maturation of chromatin compaction in cerebellar granule neurons is disrupted in *H1f2/H1f4* double knockout mice. These data raise the possibility that the neurological changes in Rahman Syndrome may arise from disrupted functions of histone H1.4 in neurons.

## Full Text Availability

The license terms selected by the author(s) for this preprint version do not permit archiving in PMC. The full text is available from the preprint server.

